# Annexins as Overlooked Regulators of Membrane Trafficking in Plant Cells

**DOI:** 10.3390/ijms18040863

**Published:** 2017-04-19

**Authors:** Dorota Konopka-Postupolska, Greg Clark

**Affiliations:** 1 Plant Biochemistry Department, Institute of Biochemistry and Biophysics, Polish Academy of Sciences, Warsaw 02-106, Poland; 2 Molecular, Cell, and Developmental Biology, University of Texas, Austin, TX 78712, USA; gbclark@utexas.edu

**Keywords:** annexin, vesicular transport, membrane trafficking, stress response, Rab protein, SNARE

## Abstract

Annexins are an evolutionary conserved superfamily of proteins able to bind membrane phospholipids in a calcium-dependent manner. Their physiological roles are still being intensively examined and it seems that, despite their general structural similarity, individual proteins are specialized toward specific functions. However, due to their general ability to coordinate membranes in a calcium-sensitive fashion they are thought to participate in membrane flow. In this review, we present a summary of the current understanding of cellular transport in plant cells and consider the possible roles of annexins in different stages of vesicular transport.

## 1. Introduction

Annexins are a multigene, evolutionarily conserved family of calcium- and phospholipid-binding proteins with a highly conserved tertiary structure among subfamilies from different kingdoms [1]. They are present in almost all eukaryotes, and prototypical proteins of this family are found in some prokaryotes [2,3,4,5]. The genomes of all plant and vertebrate species encode several annexins and the expression levels of certain annexins can be very high [3]. Annexins were first purified as minor contaminants of calmodulin purifications and were considered to be novel targets for Ca^2+^ signaling in animal cells. The contribution of annexins to plant cell adaptation to adverse environmental conditions is well documented [3,6,7,8,9,10]. Analyses confirmed that annexin 1 (ANNAT1) from *Arabidopsis thaliana* (Arabidopsis) and its homologs from different species (i.e., ANNBJ1 from *Brassica juncea*, NNANN1 from *Nelumbo nucifera*, GHANN1 from cotton, *Gossypium hirutum* and STANN1 from potato, *Solanum tuberosum*) play a role in stress tolerance of tobacco, cotton, and Arabidopsis [3,10]. In transgenic plants expressing higher levels of this annexin, degradation of photosynthetic pigments and reduction of photosynthetic activity were slower and less pronounced than in wild-type plants, and their productivity in response to different stresses was better. In some cases, ectopic expression of this annexin resulted in multi-stress tolerance [10,11,12]. Additionally, certain annexins are also able to alleviate oxidative stress in prokaryotic (bacterial) and animal cells, which strongly suggests that annexins can function via a very basic mechanisms, common across kingdoms [1]. So far, the precise molecular mechanism(s) of these biological phenomena is unknown.

Annexins are also believed to be involved in membrane-related processes, such as intracellular vesicular trafficking, endo- and exocytosis, phagocytosis and autophagy, due to their inherent ability to bind and position the membrane structures in relation to each other in a calcium-dependent manner [3,13]. The growing body of data indicates that cellular trafficking plays an essential role in plant stress responses and adaptation to changes in the environment. In Arabidopsis, undisturbed vesicle trafficking is necessary for proper signal transmission during growth and development [14], maintenance of ion homeostasis [15,16], and tolerance to salt stress [17], water scarcity [18] or in defense responses [19,20]. It was shown that osmotic stress tolerance relies on both transcriptional and non-transcriptional responses. The latter implicates regulation of stomata movements [21] and membrane permeability [22] by coordination of endo-/exocytosis of plasma membrane ion channels or trafficking of water channels, respectively. Similarly, the non-transcriptional responses of stress-related phytohormones, such as abscisic acid or methyl jasmonate are, at least partially, exerted through the changes in the polar distribution of auxin transporters from the PIN family [23]. Abscisic acid is a key regulator of abiotic stress resistance and promotes endocytosis [24] as well as sequesters auxin transporters in endosomal recycling compartments [25], whereas methyl jasmonate (MeJA) effects on PIN2 subcellular distribution varies in a concentration-dependent manner, and at higher-inhibits, while at lower-induces endocytosis [26]. Auxin used to be considered to solely function in developmental processes but recent transcriptome analysis revealed a partial overlap of expression profiles between auxin-responsive genes and stress-response genes, which suggests that auxins are also involved in stress/defense responses [27].

We speculate that plant annexins are among possible Ca^2+^ effectors that control intracellular membrane flow either by direct interaction with membrane structures or indirectly, e.g., by rearrangement of actin cytoskeleton [28]. In this context, the strongest evidence supporting this hypothesis came from the early work on mammalian annexins [29]. In this study, annexins A1, A4, A5, A6, A7 (ANXA1, ANXA4, ANXA5, ANXA6 and ANXA7, respectively) were expressed in wild-type and 13 secretory yeast mutants (*secs*) [30]. The latter included the ten *sec* mutants impaired in the late secretory pathway (LSC; *sec1*, *sec2*, *sec3*, *sec4*, *sec5*, *sec6*, *sec8*, *sec9*, *sec10* and *sec15*), two mutants defective in the transport from endoplasmic reticulum (ER) to the Golgi complex (Golgi) transport (*sec17* and *sec18*), and one mutant with pleiotropic defects (*sec14*). Yeasts lack endogenous annexins so observed effects were due to non-specific interactions between annexins and secretion machinery, and not due to complementation. None of the annexins provided full complementation of any of the *sec* mutants, but specific interactions were observed for ANXA1, ANXA6 and ANXA7 with the *sec2* mutant, and between ANXA7 and *sec4* and *sec15*. Whereas annexin A7 inhibited the growth of *sec2*, *sec4* and *sec15* mutants defective in exocytosis, ANXA1 and ANXA6 reduced the lag time associated with adaptation of *sec2* mutants to galactose-containing medium. The latter could be due to annexin-mediated correction of the defective insertion of the galactose permease into the plasma membrane (PM). Summarizing, certain annexins were able to influence specific steps in membrane trafficking associated with yeast cell growth, secretion and the plasma membrane (PM) remodeling.

The purpose of this review is to highlight the recent advances in plant membrane trafficking and consider the recent data suggesting roles for annexins in membrane trafficking. New insights into our understanding of the complex network of membrane trafficking in plant cells as well as new findings on plant annexin function are discussed.

## 2. Annexin Characteristics

**Figure 1:**
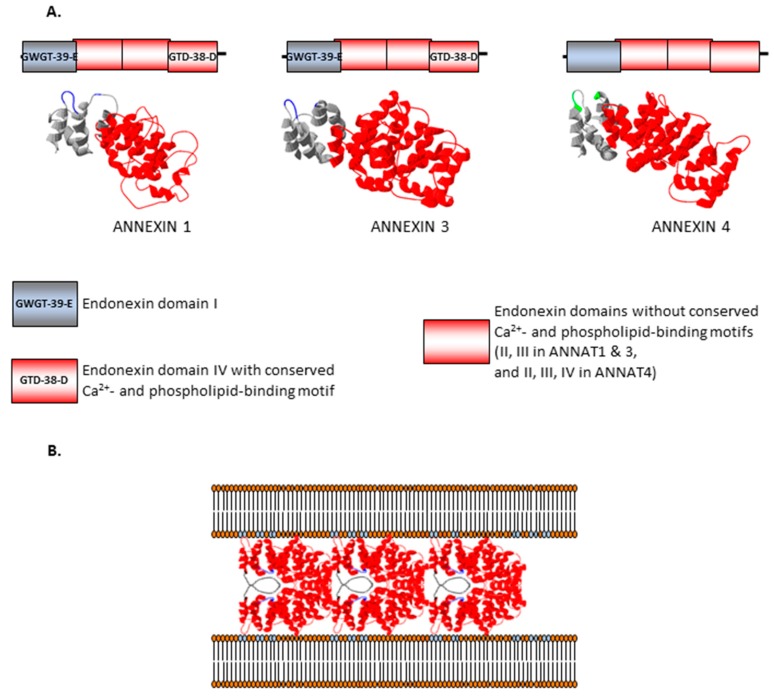
Predicted structure of three Arabidopsis annexins and proposed mechanism for annexin-membrane coordination. (**A**) Predicted structure of three Arabidopsis annexins, ANNAT1, ANNAT3, and ANNAT4. The structure was prepared with Swiss-PdbViewer, DeepView v4.1 by Nicolas Guex, Alexandre Diemand, Manuel C. Peitsch, and Torsten Schwede on the basis of existing annexin crystal structures. The overall structure of annexins is evolutionary conserved. The molecule consists of four repeats (I–IV) of approximately 70 amino acids (PFAM domain PF00191, 66 aa). In plant annexins the type II Ca^2+^- and phospholipids binding motif (GxGT-(38–40 residues)-D/E) is highly conserved in repeat I (in grey), generally lost in repeats II and III, and only moderately conserved in repeat IV (in red). In Arabidopsis, the canonical motif is present in repeat 1 of annexin 1 and 3 and a modified motif in repat IV of annexin 1 and 3. In annexin 4 there is no recognizable calcium and phospholipids binding motifs; (**B**) Possible mechanism of membrane coordination by annexins, according to [34,37]. Two opposing membranes can be coordinated by dimerizing annexin molecules. Binding to the membrane causes changes in molecular conformation and flattening of protein disc. As a result, a secondary calcium- and membrane-binding sites on the concave surface disclose, which allows positioning of the various membrane structures.

Although the primary amino acid sequences of annexins differ significantly the overall structure of proteins from this superfamily is well preserved with four well recognizable repeats (I–IV) of approximately 70 amino acids (PFAM (database of curated protein families) domain PF00191, 66 aa). Each of these repeats has the potential to have a type II Ca^2+^-binding bipartite motif, located on two different α-helices (GxGT-(38–40 residues)-D/E), but typically some of them are non-functional. In plant annexins the Ca^2+^-binding motif is highly conserved in repeat I, generally lost in repeats II and III, and only moderately conserved in repeat IV [3,13]. For example, Arabidopsis ANNAT1 and ANNAT2 have conserved Ca^2+^-binding motifs in repeats I and IV but not in repeats II and III, while ANNAT4 is more divergent (Figure 1A). In contrast, in vertebrate annexins three repeats (I, II and IV) are well preserved [1,3,13]). Each single annexin domain is comprised of 5 α-helices (A–E). Four of them (A, B, D and E) are arranged parallel and form a tightly packed helix-loop-helix bundle. In contrast, helix C is almost perpendicular and covers the remaining four on the surface [13]. The core of the helix bundle is composed largely of hydrophobic residues, while hydrophilic residues are exposed on the surface of the protein and between the domains. The tertiary structure of annexins is evolutionary conserved; a single molecule resembles a slightly curved disk with the calcium and phospholipid-binding sites located on the more convex surface and the more concave surface facing the cytoplasm. Despite the significant structural similarities responsible for their central property of Ca^2+^-dependent lipid binding, individual eukaryotic annexins are considerable specific; for example, they differ significantly in their calcium binding affinity and hence also in their membrane binding. In smooth muscle cells, annexins act as an intracellular Ca^2+^ sensors and were shown to translocate to the PM sequentially, according to their decreasing calcium affinity [31,32]. A mechanism of membrane binding was proposed which assumes that calcium ions are coordinated jointly by Ca^2+^-binding site and membrane phospholipids (membrane bridging mechanism) [33]. Accordingly, the calcium binding affinity of individual annexins has to be regarded only in relation to the composition of the interacting membrane. Membrane binding results in conformational changes and the slightly curved annexin molecule is transformed into more planar disc [34]. Such modification can reveal the secondary phospholipids binding sites on the concave surface and allows for the apposition of membrane structures [35] (Figure 1B).

Annexins are classified according to the evolutionary divisions of the eukaryotes into five families: A (ANXA, vertebrates, including humans), B (ANXB, invertebrates), C (ANXC, fungi), D (ANXD, true plants), E (ANXE, protists) [36]. The best characterized is the monophyletic A family, where 12 distinct subfamilies are encoded by 12 paralogous genes (*ANXA1*–*ANXA11* and *ANXA13*). The second, truly monophyletic clade is Archaeplastida that consist of green and red algae, and contemporary green land plants [2]. In contrast, neither fungi nor protists are regarded as a monophyletic clade and different groups within can possess and lack these proteins [2].

The possible role of annexins in membrane trafficking and secretion was originally suggested due to their ability to “annex” membranes and potentially aid in secretory vesicle fusion with the PM (Figure 1B) [3,13]. Annexins not only bind to abundant phospholipids in membranes such as phosphatidylserine but also interact with the more minor membrane phospholipids such as negatively charged phosphatidylinositols, phosphatidylglycerol and phosphatidic acid. The paradigm for annexin function is based on their Ca^2+^-dependent membrane-binding property that allows them to move from the cytosol to membranes when cytosolic Ca^2+^ levels increase in response to a stimulus. Thus annexins are viewed as dynamic, signaling proteins providing important links between intracellular Ca^2+^ signals and the regulation of various membrane functions, such as lateral membrane organization, cytoskeleton interaction with cellular membranes and membrane flow.

## 3. Membrane Flow in Eukaryotic Cells

Eukaryotic cells are subdivided by an endomembrane system into a series of discrete compartments. The diversification of the internal cellular environment enables different functions to be carried out simultaneously, such as synthesis sorting and degradation of macromolecules (proteins, lipids and cell wall precursors), and secondary metabolism. The morphology, functions, lipid and protein compositions of individual organelles are specifically designed to support individual functions. Though cellular compartments are not continuous they stay interconnected through vesicle transport systems and proteins. Lipids are constantly exchanged between different structures. In addition, membrane transport systems allow cells to stay in contact and exchange information with their environment by supporting uptake (endocytosis) and export (exocytosis) of macromolecules, particles and other chemical compounds.

In plant cells, the main organelles of the endomembrane system are the ER [38], the Golgi, comprised of numerous dispersed Golgi stacks called dictyosomes [39] and the *trans*-Golgi network (TGN) [40], multivesicular bodies/prevacuolar compartment (MVB/PVC) [41], lytic and protein storage vacuoles (LV and PSV, respectively) [42,43], the PM and the endocytic compartment [44,45]. Based on rapid labeling with the fluorescent endocytic tracer FM4-64 and morphological similarities it is generally accepted that in plants the TGN functions like the early endosome compartment (EE) in animal cells [41,46,47]. The next structure labeled by FM4-64 is the MVB/PVC which suggests that it is the plant equivalent of the late endosome compartment (LE) in animal cells. In plant cells, acidic vacuoles then function as lysosomes.

Membrane compartments communicate constantly with each other by means of transport vesicles. As in all other eukaryotes, there are two main pathways of membrane flow in plant cells: secretory and endocytic pathways [48,49]. Within the early secretion pathway (ESP), which includes the ER and the Golgi [47], vesicle trafficking occurs in two opposite directions. In forward (anterograde) trafficking, all newly synthesized proteins intended for secretion are exported from the site of their synthesis in the ER to the default destinations. In the Golgi, cargo and membrane molecules are sorted between PM/apoplast and tonoplast/vacuole lumen that collectively form the late secretion pathway (LSP) [47].

At each step of their transport, proteins can be turned back from their forward route by reverse (retrograde) trafficking. Transport within the ESP is mediated by coat protein complex II (COPII)-coated vesicles that bud off from the ER membranes and COPI-coated vesicles that are released from Golgi cisternae [50,51]. COPII-coated vesicles are involved in anterograde ER-to-Golgi transport, whereas two types of COPI-coated vesicles mediate retrograde traffic from Golgi-to-ER (COPIa) and transport between the Golgi cisternae (COPIb) [50]. A third type of vesicle, clathrin-coated vesicles (CCV), operates within the LSP and supports both forward transport and delivery of endocytosed material to the endosomes. The second distinct secretion route supports delivery of the precursors of cell wall polysaccharides synthesized in the Golgi [52,53]. Cellulose is the only component of the cell wall that is produced in situ at the cell surface by the PM localized enzymes, whereas hemicelluloses and pectin precursors are synthesized in the Golgi and only then are transported to the cell surface and assembled into polymers [54,55,56]. It is estimated that up to 80% of the metabolic activity of the Golgi in plant cells is engaged in polysaccharide synthesis. In the ESP, retrograde traffic counterbalances the continuous forward flux, and enables the cell to maintain the size of the different compartments as well as to recycle components of the resident transport machinery to the appropriate compartment and prevent loss of resident proteins captured by coincidence from donor compartment [57].

Much less is known about the LSP. The TGN is the first site where the biosynthetic/secretory and endocytic pathways intersect [49]. At the TGN, secretory trafficking could potentially branch out toward the different post-Golgi compartments, i.e., PM or PVCs/MVBs [40,57,58]. Proteins with no sorting signal/signals are transported to the PM as a default. Other proteins carrying vacuole sorting signal/signals (VSSs) are recognized by the appropriate vacuolar sorting receptor and transported into PVCs though there are also some indications that recognition of cargo by VSRs may occur already in the ER [59,60,61,62]. Receptors and membranes are returned to the TGN for the next cycles of delivery. Trafficking within the LSP is mediated mainly by clathrin-coated vesicles (CCVs). These vesicles are formed at the PM during endocytosis and at the surface of the *trans*-Golgi network, and they support receptor-mediated post-Golgi trafficking as well as endocytic protein transport.

**Figure 2:**
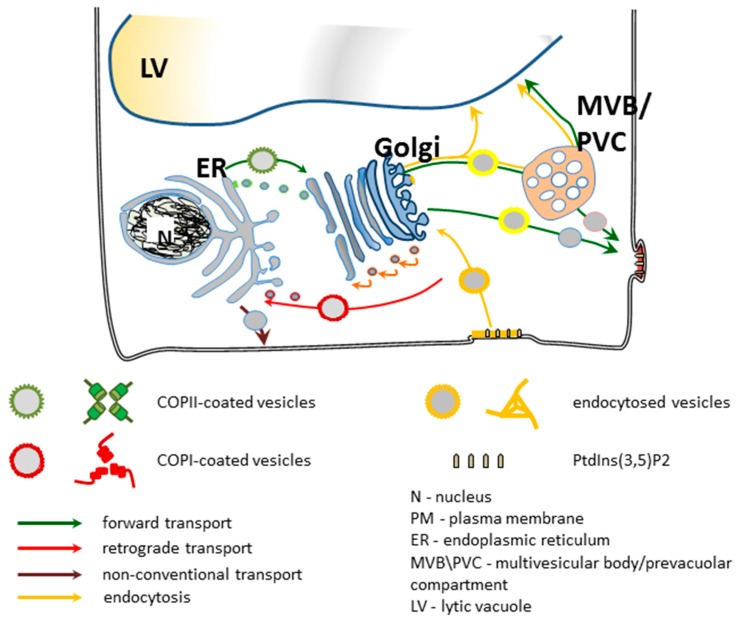
Model depicting intracellular transport in plant cells.

Trafficking of soluble cargo to the vacuoles occurs not only by the TGN but also via other routes that have recently been described. There is evidence showing that vacuolar proteins can bypass the Golgi (non-conventional secretion pathways) [63], or first reach the PM and only then come back to the vacuole, because undisturbed endocytosis is required for proper development of vacuolar system [64]. Additionally, an intermediate compartment between the PVC and the LV was described in the leaf epidermis of tobacco (*Nicotiana tabacum*) [65], where proteins can be stored for various lengths of time before reaching their final destination. Moreover, additional non-Golgi secretion pathways of leaderless proteins are still being discovered [66]. A schematic depiction of membrane trafficking in plant cells is shown in Figure 2.

In silico analysis predicts that more than 17% of all Arabidopsis proteins enter the endomembrane system to be transported via the Golgi to the different default designations [67]. Proteins having N-terminal leader sequences are co-translationally inserted into ER lumen through the SEC61 pore complex [68] where they subsequently undergo *N*-glycosylatation. This modification recruits enzymes required for the proper folding of a nascent polypeptide and the machinery responsible for quality control [69]. Properly folded proteins are either retained within the ER or concentrated at discrete domains of the ER exit sites (ERES) specialized for secretion and then incorporated into nascent COPII-coated vesicles for delivery to the Golgi [70]. In the Golgi, proteins undergo final maturation and are sorted to the vesicles traveling to the appropriate default localization (apoplast/PM or tonoplast/vacuole) based on the presence of the short amino acid signaling sequences (sorting motifs). Transport between the ER and the Golgi requires energy and small monomeric GTPases from the ARF family. The very last Golgi cisterna in the TGN is also a site where forward and retrograde protein flows meet. It functions as a sorting station at the crossroads of the endo- and exocytic pathways. Endocytic retrograde route enables both retrieval of trafficking machinery and degradation of already dispensable membrane proteins. Correct targeting of vesicles relies on the presence of specific tags on the donor compartment and fitting tags on the vesicles. Fusion of Golgi-derived vesicles carrying newly synthesized vacuolar proteins and from early/recycling endosomal compartments generates multivesicular body (prevacuolar compartment, MVB/PVP). Within MVB/PVC, two kinds of sorting processes occur—the recycling of vacuolar cargo receptors mediated by the retromer complex and the sorting of PM protein into internal vesicles by the ESCRT machinery.

Undisturbed membrane trafficking is of key importance for maintaining homeostasis and stress responses in all eukaryotic organisms. Mutations in membrane trafficking proteins that block secretion are often lethal. While not lethal, many annexin loss-of-function mutants are sensitive to stress and their corresponding gain-of-function mutants are stress tolerant. These phenotypes may be due to their antioxidant activity, Ca^2+^ transport activity and and/or their role in regulating membrane trafficking. In yeast (*Saccharomyces cerevisae*) mutants without a vacuole are viable despite severe biogenesis defects [71,72]. In contrast, in plants, vacuoles are essential for development and annexins can associate with the vacuolar membrane at physiologically relevant Ca^2+^ levels. Usually mutations that disrupt their development and/or morphology, such as the Arabidopsis *vacuoleless* (*vcl*)/*vps16*, are embryo lethal [71,73]. As well, mutations that disrupt single syntaxin genes (protein that form a SNARE complex executing membrane fusion) are frequently lethal at the phase of gametophyte or seedlings development [14,74,75,76,77].

### The Role of Calcium in Plant Membrane Trafficking

The role of Ca^2+^ in intracellular membrane trafficking is still poorly characterized. For a long time it has been known that in animal cells transient elevations of Ca^2+^ ions in the cytosol triggers the fusion of secretory granules and synaptic vesicles with the PM. But in fact, membrane fusion occurs many times during the secretory pathway, not only at the PM, so Ca^2+^ could potentially affect all these steps.

The best recognized model of the Ca^2+^-regulated membrane trafficking event is neuronal SNARE-mediated exocytosis that supports quantal release of neurotransmitters. As such, SNAREs are not Ca^2+^ sensitive proteins and assembling of *trans*-SNARE complexes at the interface between synaptic vesicle and plasma membrane as well as subsequent fusion of bilayers are basically not calcium-dependent processes. Calcium responsiveness is conferred by an additional interactions with Ca^2+^-binding proteins, such as synaptotagmins or calmodulins [78,79,80] that are activated in different but overlapping ranges of [Ca^2+^]. Synaptotagmins ensure the synchronization of Ca^2+^-dependent exocytosis with the presynaptic action potential [78]. The effect of calmodulins is much more complex, as they interact with multiple target proteins implicated in exocytosis, e.g., individual Soluble NSF Attachment Protein Receptor (SNAREs; e.g., VAMP2, AtSYP13 [79,81], Ca^2+^ channels, Ca^2+^/calmodulin kinase II [82,83,84], Rab3A [82,84,85] and Munc13 [85,86]). While doing so, calmodulin and synaptotagmin can cooperate to define the range of concentrations, in which exocytosis occur.

With the accumulation of more data it has become clear that Ca^2+^ is a basic cofactor required for fusion of different biological membranes [87,88,89,90,91,92,93]. The constitutive secretory pathway seems to be a mosaic of Ca^2+^-dependent and Ca^2+^-independent processes. Addition of membrane-permeant chelators to intact, living cells results in inhibition of both anterograde and retrograde transport, but subsequent transport steps were affected to different extents [94]. The initial step of the secretory pathway, i.e., COPII vesicle fusion, was not inhibited by Ca^2+^ chelators, while the second step, trafficking between the ER and Golgi intermediate compartment (ERGIC) and within the Golgi was inhibited. Downstream of the Golgi, there were no retention points between the PM. As well, endocytosis was not impaired, while endosome-to-Golgi and Golgi-to-ER trafficking were blocked [89].

Collectively, the data described above strongly suggests that one of the possible mechanism of calcium action is modulation of activity of transport-related proteins, such as annexins, small GTPases of RAB family or vesicle coat proteins. Besides Rab3A in neurons the activity of Rab11a in contractile vacuoles of *Dictyostelium discoideum* was shown to be controlled by the targeted Ca^2+^ release through an ion channel P2XA [95]. In pneumocytes, Ca^2+^ entry via vesicular P2X4 channels was reported to promote the opening of fusion pores and release of the vesicle content [96]. Thus, the calcium-dependent regulation of Rab proteins may be a common phenomenon, but the range of this mechanism remains an open question. P2X receptors are also expressed on intracellular membranes in some cell types of multicellular organisms, though they are not found in higher plants [97]. However, treatment of plants with extracellular ATP (eATP) induces Ca^2+^ influx in plants [98,99,100,101,102] and eATP regulates growth in a variety of plant cells and tissue [103]. Recently a lectin kinase receptor was identified as the first plant eATP receptor [104]. Interestingly, eATP-mediated Ca^2+^ release in response to salt treatment was impaired in an Arabidopsis annexin mutant [105].

Calcium was also found to stabilize of COPI/COPII vesicle coat. ALG-2 (apoptosis-linked gene 2) is a Ca^2+^-binding protein that acts as a Ca^2+^ sensor on the membrane of ER export sites where COPII coated vesicles form and the presence of Ca^2+^ the association of the sec31 subunit with the membrane is stabilized [106]. The Ca^2+^ sensor for COPI retention on the Golgi has not been identified yet but the yeast equivalent of the COPI coat tethering at the Golgi membranes requires, among other proteins, the Rab GTPase, ScYPT1 and the USO1 protein [107]. The latter is homologue of golgin p115 [108], which tethers Rab1 to COPI vesicles to the *cis*-Golgi. A single point mutation in ScYPT1 (*YPT1*^Ile121^) results in temperature-dependent dominant-lethal phenotype in mutant cells. A mutation in SLY1 results in the elevation of Ca^2+^ levels in the cytosol and suppresses this phenotype [109,110]. Ca^2+^ does not substitute for the specific USO1- or YPR1-dependent tethering process, instead, it bypasses the need for efficient vesicle tethering.

A second possible mechanism of Ca^2+^ action on membrane fusion can be attributed to the physical interaction of the ions with acidic phospholipids such as phosphatidylinositol phosphates resulting in modification of the physical properties of bilayer. In model membrane fusion systems calcium ions increased the rates of lipid mixing and promoted the formation of fusion pores [111]. Interaction can also reduce the energy barrier of membrane clustering to produce hemifusion state (fusion of only external leaflets of two lipid bilayers) and then stabilize highly curved membrane. Depending on lipid composition and [Ca^2+^]_cyt_, their interaction can promote negative or positive membrane curvature, which in turn influences fusogenicity of many biological membranes [112].

Finally, the Ca^2+^-mediated regulation of membrane fusion events can have an indirect effect, such as stabilizing of COPI and COPII coats [113] or inducing re-arrangement of actin microfilaments [114,115,116,117]. Proper coat assembly is required to form the vesicles themselves and to select cargo as well as for biogenesis of the ESP compartments [89,118,119]. Actin microfilaments create tracks for secretory vesicle movement and actin polymerization-driven processes may control vesicle budding and movement of endosomes, so Ca^2+^ can also regulate endocytosis [120]. In animal cells, fusion of endosomes and lysosomes is controlled by transient increases in cytosolic Ca^2+^ ions and the action of two downstream effectors, calmodulins [88,90] and Rab GAP proteins [95].

Less is known concerning such processes in plant cells but the high level of evolutionary conservation of overall tertiary structure strongly supports the likelihood that annexins may also function as Ca^2+^ effectors regulating intracellular membrane flow in plants. Additionally, direct measurements of vesicle fusion using patch clamp techniques in a single aleurone protoplast revealed that exocytosis in plants is also a Ca^2+^-dependent process [111]. Secretion of individual proteins was also shown to be a Ca^2+^-dependent process (e.g., inducible secretion of peroxidases in order to loosen cell wall to enable cell elongation) [121]. Finally, a Ca^2+^ gradient in polarized cells contributes to the control of secretion of cell wall material [122].

However, some differences in how plant annexins regulate membrane flow, resulting from different cytoarchitecture and physiology, should be expected. In plant cells the ER is a not a major intracellular Ca^2+^ store as it is in animal cells. Because the large central vacuole and apoplast in plants also participate in Ca^2+^ storage [123], Ca^2+^ signaling is spatially different in plant cells [87]. Plant secretion pathways also have several unique features distinguishing it from comparable routes in animal cells, so in the following section we will summarize the status of current knowledge about plant membrane trafficking.

## 4. Annexins in Membrane Trafficking

### 4.1. What Is Known from Vertebrate Annexins

Since their discovery in animal cells, one of the earliest and most often suggested function for this protein family has been a participation in the process of secretion. To the extent that the function of annexin in plants might parallel its function in animals, it is worth considering the reports of animal annexin participation in membrane trafficking and endocytosis. In the early studies the best evidence for a role in secretion was for ANXA7, originally known as synexin. It was postulated to participate in secretion due to its membrane fusion properties promoted by Ca^2+^ and GTP [124,125]. It promoted very vigorous aggregation of yeast secretory vesicles in vitro, in contrast to ANXA2, which was only weakly active in the yeast vesicle aggregation assay [29]. Another annexin, ANXA13b, was found to function in apical secretion by association with lipid microdomains [126]. Besides the PM in BHK cells there are also all other membrane compartments, such as nuclear envelope, the ER, the Golgi, the plasma membrane, early endosomes (EE), late endosomes (LE) and lysosomes was clearly identified as a target for annexins binding [127]. Later on, there is also a role for annexins in the endocytic pathway [128] including intracellular positioning of recycling endosomes [129] and the biogenesis of multivesicular body (MVB) [130,131]. ANXA2 was also shown to be required for establishing cell polarity, cytokinesis and endocytosis in HeLa cells [].

Beyond these original studies, evidence that certain animal annexins function in different stages of membrane trafficking has continued to be obtained. Over a dozen annexins can be expressed in a single mammalian cell forming a sophisticated Ca^2+^-sensing network and are able to bind to membranes in a concentration-dependent manner, beginning from the most sensitive ANXA2 followed subsequently by ANXA6, ANXA4 and ANXA1 [132,133]. Moreover, after induction they are translocated to distinct membranes [31,32]. ANXA1 and ANXA2 were translocated to the endosomal membrane, with ANXA2 additionally being involved in intracellular vesicle movements. ANXA5 was associated with the LE and the Golgi, ANXA6 with the Golgi, vacuolar membranes and the ER. ANXA1 and ANXA5 localized to the nucleus. These results suggest that particular annexin is functionally specialized to control individual set of subcellular membranes in response in diversified manner. Ca^2+^ is known to regulate different aspects of secretion and vesicle trafficking, beginning at the very initial stages-transport from the ER to the Golgi up to the fusion with the PM and endocytosis. However, the role of Ca^2+^ in the early stages of secretion is still poorly understood, especially relative to the rather extensive knowledge about its function in the different phases of the LSP beyond the Golgi, such as vacuolar transport, exocytosis, endocytosis, recycling of membrane components. There is now evidence that Ca^2+^ helps to regulate secretion and vesicle trafficking at the different stages of vesicular trafficking by means of the interaction with annexins. Within the ESP, ANXA11 was shown to regulate the ER-to-Golgi transport by stabilizing the SEC31A protein (a component of an outer cage of COPII-coated vesicles) at the ER [113]. ANXA2 is found at exocytotic sites in chromaffin granules and is needed for Ca^2+^-dependent formation of lipid microdomains essential for exocytosis in these cells [134]. Decreased expression of ANXA2 results in inhibited exocytosis in chromaffin granules. More recently, another study found that binding of ANXA2 to membranes induces formation of microdomains enriched in cholesterol and phosphatidylinositol 4,5-bisphosphate [PtdIns(4,5)P_2_] and also inward vesicle budding in giant unilamellar vesicles [135]. Finally, it was shown that the ANXA2 was able to partially restore Ca^2+^-dependent secretion in digitonin permeabilized chromaffin cells [136]. A key role in the events leading to exocytosis was assigned to a 16 amino acid peptide (P16), corresponding to C-terminal end of ANXA2 (shared with 14-3-3 proteins). The partial effect of P16 on secretion under a variety of experimental conditions suggests that the annexin is not essential for exocytosis but only regulate its extent possibly by establishing in a calcium-dependent manner protein-protein interactions [137]. There is both in vitro and in vivo evidence that ANXA7 regulates catecholamine release from stimulated chromaffin cells and the BoNT type C-mediated inhibition of membrane fusion relies on the cleavage of ANXA7 [138]. Animal annexins were also recently found to be involved in endocytic trafficking [139]. ANXA2 facilitates endocytic trafficking of antisense oligonucleotides used as tools in this research [140]. Recent studies also revealed involvement of the ANXA1 in membrane trafficking events [141,142]. ANXA1 and ANXA2 were shown to participate in retrograde trafficking [143,144] and annexin A2 was proposed to play a role in the biogenesis of MVBs [131]. A similar study showed that ANXA1 was required for EGF-stimulated inward vesiculation in multivesicular endosomes [145]. Two mechanisms of endosomes fusion was proposed, one that relay on calcium and annexins, and another that is calcium independent [146].

Finally, abundant studies suggest that annexins are important for membrane repair. This process is induced by uncontrolled influx of extracellular Ca^2+^ and requires intact membrane trafficking [147]. Depending of the type of membrane injury different mechanisms of membrane repair have been described, including: (i) Ca^2+^-activated, homotypic vesicle fusion and patch formation, which undergo exocytosis [148]; (ii) endocytosis of the membrane regions permeabilized by pore forming toxins or mechanical wounded [149,150]; (iii) blebbing, formation of the protrusions from the damaged membrane segments locally detached from cortical cytoskeleton with adjacent cytoplasm [151]. Annexins are able to induce aggregation and fusion of intracellular vesicles/lysosomes and thus can assist exocytosis and offer a scaffold for endosome formation. ANXA1 and ANXA2 contribute to membrane repair by aggregation and fusion of intracellular vesicles [152]. The blebs are sealed off from the cell body by plugs of ANXA1 [151]. ANXA5 in turn can form a two-dimensional network beneath the injured plasma membrane [153]. Finally, ANXA6 assemble a “cap” on the membrane repair patch [154].

### 4.2. Plant Annexins

All of the contemporary families of plant annexins originate from one to three founding members in mosses and ferns [3]. The main expansion of annexin genes in plants occurred at about 450 mya during colonization of the more challenging drought-prone land environment, before the divergence of monocots and dicots [155]. Thus far, more than 400 plant annexins have been identified and based on primary amino acid sequence grouped in 17 phylogenetically related subfamilies. Just as their animal counterparts plant annexins form multi-member families in a respective species (8 in Arabidopsis [156]; 9 in rice *Oryza sativa* [157]; 12 in maize *Zea mays* [158] and potato *Solanum tuberosum* [10]; 11 in *Solanum lycopersicum* [159]; and 23 in soybean [158]).

Plant annexins were first purified based on their ability to bind phospholipids/membranes in a Ca^2+^-dependent manner. They have been found to be associated with vacuolar, nuclear and plasma membranes as well as the Golgi and Golgi-derived vesicles [160,161,162]. Results suggesting annexin involvement in plant secretory processes first came from early localization studies. Using immunological approaches annexins were located at the tips of polarly growing cells such as pollen tubes and fern rhizoids [163,164]. Further, immunolocalization studies in peas found high levels of annexin immunostain in other highly secretory cell types, such as young, developing xylem cells and outer root cap cells [160]. In this study, immunogold localization showed annexin association with the *trans*-Golgi membranes, Golgi-derived secretory vesicles and PM. It is noteworthy that the level of immunogold labeling of annexin in root cap cells greatly increased as the root cap cell progressed toward the periphery transitioning into highly secretory outer root cap cells. Another early study also showed that plant annexins, like animal annexins, can induce aggregation of secretory vesicles in vitro [165].

The results from the early studies were only suggestive of a role for plant annexins in secretory processes. However, in two landmark papers, a maize annexin was demonstrated to induce aggregation of secretory vesicles [165] and to directly have a positive effect on exocytosis in root cap protoplasts [166]. The addition of annexin protein promoted Ca^2+^-dependent secretion of polysaccharides from root cap cells, while anti-annexin antibodies blocked this ability. Interestingly, addition of GTP inhibited secretion in the root cap protoplast cells.

The results from more recent studies have provided evidence that plant annexins play an important role in abiotic and biotic stress responses. Ectopic expression of Arabidopsis ANNAT1 and its homologs confers tolerance to drought, osmotic and salt treatments as well as tolerance to pathogen attack. These stress tolerant phenotypes observed are likely due to the antioxidant activity associated with this annexin. This protein activity persists across biological kingdoms [12,167,168,169,170,171,172]. For example, expression of ANNAT1 homologs can limit lipid peroxidation levels induced by stress treatments. The precise mechanism of action for plant annexin antioxidant activity is still unclear, however certain animal and plant annexins have conserved redox-sensitive cysteine residues, such as Cys-8 in mammalian ANXA2 [173,174]. Irreversible inhibition of this cysteine by *N*-ethylmaleimide treatment did not interfere with phospholipid binding, however it abolished in vitro liposome aggregation. Cys-8 can possibly undergo repeated redox cycles and after oxidation by ROS it can subsequently be reduced by the thioredoxin system [174]. Indeed, compared to the control cells ANXA2 depleted cell lines accumulated higher levels of ROS, as well as displayed increased activation of the oxidative stress-induced proapoptotic kinases (p38, JNK, AKT) and a higher level of cell death [174]. Plant annexins may utilize a similar mechanism, possibly even more so as they possess multiple reactive cysteines [6]. Interestingly, ectopic expression or overexpression of certain plant annexins also induces changes in gene expression [12]. This observation suggests the possible Ca^2+^-induced translocation of annexin to the nucleus [161,174,175,176,177]. However, as discussed earlier annexins could also function in stress responses by regulating endocytosis and exocytosis, which is known to play a critical role in plant stress responses.

There are also a number of biochemical properties found in plant annexins that could be important for regulating endocytosis and exocytosis processes, including F-actin binding, modulation of Ca^2+^ influx activity and association with lipid microdomains. Plant annexins from different species have been shown to bind F-actin in vitro [178,179,180], and some have an IRI motif found in their fourth repeat that is potentially responsible for actin binding. There is a review highlighting the importance of annexins in regulating actin filament organization and dynamics in plants [181].

Ca^2+^ is a critical signal in directing polarity in plant cells, and membrane trafficking is one important component for establishing and maintaining cell polarity [182]. While it is still controversial whether some plant annexins act directly as Ca^2+^-permeable channels, it is clear that certain plant annexins modulate Ca^2+^ influx. For example, ANNAT1 has been shown to facilitate Ca^2+^ influx in response to H_2_O_2_, which can regulate growth [9,183]. Certainly their presence at the tip of polar growing cells suggests a possible role in regulated Ca^2+^ influx during cell expansion.

Association with lipid microdomains is an important component of annexin-mediated exocytosis in animal cells. These microdomains are detergent-resistant membrane fractions, and plant annexins have recently been identified in such fractions [184]. Although these in vitro findings do not necessarily indicate that annexins function in membrane microdomains in vivo, some initial experiments on the transient expression of ANNAT1 in *Nicotiana benthamiana* showed that it co-localizes in situ with a lipidated fluorescent protein that is specific for sterol-enriched membrane microdomains [185].

However, in vivo, there may be exceptions to this paradigm for annexin function as certain annexins are able to bind to membranes in a Ca^2+^-independent manner and some annexins are also found in the apoplast, e.g., ANNAT1 [186].

### 4.3. Plant Annexins in Membrane Trafficking—Where We Are Now

There are eight members of the annexin gene family in the model plant, Arabidopsis (ANNAT1–8). Studies on these annexins have demonstrated that different ones have both distinct and overlapping tissue- and developmentally-specific expression patterns as well as different sub-cellular localization [156]. There are also data suggesting that certain plant annexins are multifunctional. The best example of such protein is ANNAT1, the most abundant and well-studied annexin in Arabidopsis. It and its homologs in other plant species have been shown to promote Ca^2+^ influx as well as have antioxidant activity. It also appears to play important roles in seed germination as well as abiotic and biotic stress responses [3,7,187].

Data collected so far showed that in Arabidopsis cells individual annexins has a potential to regulate membrane events in different cellular compartments (Figure 2). ANNAT1 has been found in the PM proteome [188], while ANNAT3 was found in the tonoplast proteome [189]. Another Arabidopsis annexin, ANNAT4, was shown to interact with two sets of Qa SNAREs located at PM (AtSYP121, AtSYP122, AtSYP123) and at the PVC/tonoplast (AtSYP21 and AtSYP22) [190]. Localization studies indicate that ANNAT1 may also function in secretion. There is a strong correlation between the immunolocalization patterns for ANNAT1 and ANNAT2 and the secretion of polysaccharides, as assessed using ^3^H galactose in young seedlings [164]. There are also clear differences between ANNAT1 and ANNAT2 in their localization patterns: ANNAT1 is located at the cell periphery of root epidermal cells, root hairs and root cap cells while ANNAT2 is located at the cell periphery of hypocotyl and cotyledon epidermal cells. These results suggest that ANNAT1 and ANNAT2 can regulate Golgi-mediated secretion of polysaccharide precursors to the PM.

Two other Arabidopsis annexins, ANNAT3 and ANNAT5, have also been implicated in membrane trafficking. In the case of ANNAT3, recent evidence suggests it has a direct role in post-Golgi vacuolar transport [191]. Another study utilizing RNAi found that knock-down of ANNAT5 expression in Arabidopsis pollen resulted in severe sterility [192]. In the case of ANNAT5, gain-of-function mutants expressing higher levels of this annexin were resistant to brefeldin A (BFA)-induced inhibition of pollen germination and pollen tube elongation [193]. In fact, there was a positive correlation between the level of ANNAT5 expression and its ability to block BFA effects on pollen. This finding may be related to the observation that BFA-treatment of pollen has multiple effects on membrane trafficking. It promotes Golgi-mediated secretion but inhibits endocytosis [194], disrupts endomembrane trafficking by negatively affecting the formation of a specific subset of endosomes, and indirectly blocks actin polymerization at the pollen tube apex [195].

Because ANNAT5 was also shown to bind actin, the pollen of ANNAT5 gain-of-function mutant was treated with lactrunculin B (LatB) [193], which blocks actin polymerization thus inhibiting pollen germination and pollen tube elongation. Their results indicated that higher levels of ANNAT5 were not able to overcome LatB-mediated effects on pollen. So, although ANNAT5 appears to regulate endomembrane trafficking in pollen in a Ca^2+^-dependent manner, the exact mechanism(s) still needs to be determined.

Cotton fiber elongation is mediated by both diffuse and polar growth. The first annexin identified in cotton was shown to associate with callose synthase and potentially regulate the activity of this enzyme [196]. More recently, two cotton-fiber annexins, GHANN2 and GHFANNX, have been shown to be important in fiber elongation. RNAi silencing of GHANN2 inhibits fiber growth as well as Ca^2+^ influx at the tip of expanding fibers, which could be required for Ca^2+^ directed maintenance of polar secretion [197]. GFP-tagged GHFANNX was located on the peripheral and cytoplasmic side of the apex of cotton fiber tip, and the authors suggested this annexin can be associated with Golgi-derived vesicles in this region [198]. GHFANNXA was found to promote Ca^2+^ influx and cause reorganization of actin filaments in cotton fibers, two mechanisms that could potentially affect delivery of cell wall components needed for expansion [199].

## 5. Secretory Trafficking Pathway

For decades the secretory pathway in eukaryotic cells has been an object of intense study. The current knowledge of this processes in plant cells is summarized in Figure 2. Despite intense efforts, many aspects still remain unclear. In plants transport of proteins between the ER and the Golgi does not rely on cytoskeleton but is energy dependent. Two mechanisms of ER-to-Golgi transport of newly synthesized proteins were proposed: non-selective bulk flow and cargo capture [200,201]. In principle, they are not mutually exclusive but rather cooperate to different extents, depending on the type of protein [202]. For bulk-flow the basic protein machinery for ongoing vesicle traffic is sufficient and no additional factors are needed. In plant cells, it is efficient enough to maintain the rate of soluble protein transport through the secretory pathway [203]. In contrast, cargo capture is a selective process and requires both the presence of sorting signals on proteins and specialized protein machinery to carry out segregation and cargo concentration on the donor compartment. In this case, cargo is concentrated before exiting the donor compartment.

Protein loading and vesicle transport among different membrane compartments rely on action of small monomeric GTPases. The Arabidopsis genome contains 93 genes that encode small GTPases [204]. The high level of evolutionary conservation of GTPases in eukaryotes implies their significance in cellular signaling processes [205]. Among them Rab, Rho and Arf GTPase families function in distinct steps of membrane trafficking, from formation of vesicles on donor membranes, to directing trafficking specificity, and ending with facilitating of vesicle docking and fusion with the target membranes [206,207,208]. Arabidopsis Arf GTPase family has 21 proteins, and its members mediate the assembly of several sets of coat protein complexes [204]. For example, AtARF1 facilitates the assembly of COPI-coats and the AtSAR1 mediates the ER-to-Golgi transport by COPII-coated vesicles. The second family of proteins that determine organelle identity and provide specificity for targeted membrane fusion events SNARE families [83,208,209]. RAB GTPases promote the initial docking, whereas subsequent fusions of vesicles and target membranes is executed by SNAREs. SNAREs form a complex in which 3 glutamine-bearing proteins (Qa, Qb, Qc) and one arginine (R) component are necessary to drive a membrane fusion [210]. In vivo a functional SNARE complex is formed only by the cognate SNARE partners, though in vitro as long as a member of each subclass is present there is no discrimination between cognate and non-cognate sets [211]. Apparently, in plant cells the specificity is achieved through the presence of additional mechanisms, possibly through the action of proof-reading proteins such as SM-proteins, and/or by lateral segregation of SNAREs at the contact site of fusing organelles [212]. In Arabidopsis there are 54 SNARE genes, including 18 Qa-SNAREs (syntaxins), 11 Qb-SNAREs, 8 Qc-SNAREs, 14 R-SNAREs (VAMPs) and 3 SNAPs (Qb/Qc) [213]. Most of them are located on specific intracellular compartments: 6 in the ER, 9 in the Golgi, 4 in the TGN, 2 in endosomes, 17 on the PM, 7 in PVC/vacuoles, 2 in TGN/PVC/vacuoles, and 1 in TGN/PVC/PM.

Post-Golgi transport is directed to the PM for secretion or to the tonoplast for vacuole deposition. Along with secretory routes there is endocytic traffic and both retrieval of trafficking machinery and degradation of already dispensable proteins is ongoing. Therefore, tightly regulated molecular mechanisms are required to coordinate multidirectional vesicle movement. Correct vesicle targeting relies on the presence of specific tags on the donor compartment and fitting tags on the vesicles. The first selection step is mediated by selective cargo sorting to the different types of vesicles in the donor compartment. It is achieved by interactions of specific sorting signals belonging to the cargo molecules with membrane receptors and coat proteins in the cytosol. Immunolocalization data suggests that annexins are good candidates for participating in the last secretory pathway in plant cells.

After vesicle budding and uncoating, the molecular mechanisms enabling the precise direction of trafficking vesicles to their appropriate destinations rely on differential labeling of target membranes. This is achieved by specific lipid composition, such as the previously mentioned increased content of sterols and thickness along the secretory pathway, and enrichment in specific molecular species of minor lipid components synthesized in situ, such as phosphatidylinositol (PtdIns). ANXA2 was shown to be important in the formation of membrane domains at the site of exocytosis in animal cells as well as was also found on clathrin-coated vesicles [214] and contains two motifs for interacting with clathrin recruiting proteins [215]. These observations led to the suggestion that ANXA2 could be responsible for coupling of exo- and endocytosis events [216]. Based on these results, plant annexins may be expected to also modulate exo- and endocytosis via lateral organization of membrane microdomains. Endo- and exocytosis need to be balanced and negatively charged phosphoinositides (PIs, phosphorylated derivatives of phosphatidylinositol) regulate both these processes. As will be discussed later in this review, in addition to annexins there are other trafficking proteins that can bind PIs such as certain epsin and clathrin adaptor proteins.

Phosphatidylinositol and its phosphorylated forms are minor nonstructural components of biological membranes present mainly on the cytoplasmic leaflet of the PM. Their transient occurrence due to a high rate of metabolic turnover suit them well to regulate a wide variety of cellular processes, including different aspects of membrane trafficking [28,217]. In mammalian cells, PIs are implicated both in the exocytosis [218] and endocytosis [219,220]. PIs seems to exert their functions via various mechanisms. First, they are able to recruit specific cytosol proteins that recognize specific head groups protruding from the plane of membrane into the cytosol via specialized protein domains [221,222,223]. A number of proteins regulated by PI-binding have already been identified. During clathrin-mediated endocytosis (CME) PIs-binding proteins, such as AP-2, AP180, epsin, and dynamin coordinate the recruitment of vesicle coat proteins [224]. Second, due to inverted conical shape [225] local accumulation of PIs can affect biophysical properties of membranes facilitating development of areas of increased membrane curvature [226,227]. Consequently, PIs are able to simultaneously stabilize transient stages of secretory vesicles fusion with the plasma membrane during Ca^2+^-triggered exocytosis and vesicle budding towards the cytosol during endocytosis [228]. It was found that the level of phosphatidylinositol 4,5-bisphosphate (PtdIns(4,5)P_2_) determines the rate of vesicle priming, the size of the readily releasable vesicle pool, and ongoing rates of exocytosis in stimulated cells [227] as well as regulate SNARE dependent fusion [229]. Third, PIs can act as precursors for the formation of soluble inositol polyphosphates.

There are remarkable differences in the spectrum of phosphorylated PI-based species between plants and other eukaryotes. Only five of the seven known PI species have been detected, namely the phosphatidylinositol monophosphates: PtdIns3P, PtdIns4P and PtdIns5P, and the phosphtidyloinositol bisphosphates: PtdIns(3,5)P_2_ and PtdIns(4,5)P_2_ with PtdIns4P being the most abundant [230], while PtdIns(3,4)P_2_ and PtdIns(3,4,5)P_3_ have not been detected yet in plants. Detailed information on the molecular targets of PIs is still not available. The results obtained so far indicate that PtdIns are involved in the regulation of the central machinery for membrane trafficking and protein sorting. Analysis with fluorescent probes specific for particular species of PtdIns revealed that PtdIns(4,5)P_2_ is predominantly present in the PM at the tip of growing root hairs upon salt stress, PtdIns3P in motile membranous structures, or tonoplast and PtdIns4P in the TGN and the PM [231,232,233]. This distribution patterns at least to some extent coincide with the subcellular localization of the individual kinases catalyzing PI-synthesis and the phenotypes of the respective knock-out mutants [234,235]. Overall, the general conclusion is that the delivery/retrieval of vesicles to and from the PM is accompanied with a progressively increasing degree of phosphorylation (PtdIns→PtdIns4P→PtdIns(4,5)P_2_) in subsequent membranes [234]. It appears that PIs in plants are also involved in controlling the central machinery for membrane trafficking and protein sorting. Thus, they affect essential processes, such as the establishment of cell polarity or cell wall deposition during plant growth, development [234] and environmental responses [236].

Although in biological membranes the preferred phospholipid ligand of annexins is phosphtidylserine certain annexins also have affinity for other anionic phospholipids, among them particular PI. For example, ANXA2, ANXA8 and possibly ANXA1 display a calcium-enhanced affinity for phosphatidylinositol-4,5-bisphosphate, though for the latter the data are conflicting [237,238,239,240]. Despite lack of a well-defined PtdIns-binding domain in annexins, this interaction is direct and specific. Binding of ANXA2 to PtdIns(4,5)P_2_ induces formation or stabilization of actin assembly sites at cellular membranes [238,239]. PtdIns(4,5)P_2_ is localized in cholesterol-rich membrane microdomains in the PM [241], and ANXA2 was shown to cooperatively bind to cholesterol- and PtdIns(4,5)P_2_ containing bilayers [135], which suggests that ANXA2 can also be implicated in lateral organization of membranes [242]. The selectivity of plant annexins toward PIs has not been analyzed experimentally yet, but the similarity in the overall structure among plant and animal annexins strongly suggests that there can be similarities in their mode of action.

### 5.1. Between Golgi and Plasma Membrane: Forward Route and Exocytosis

Soluble secretory proteins without sorting signals are secreted by default [243,244]. Similarly, membrane proteins that enter the secretory pathway are directed to the PM unless they have some tag/tags exporting them to the vacuoles [66,245]. Analysis of the transport of fluorescently labeled protein revealed that traffic between the Golgi and the PM is direct and rapid, without any specific compartments in-between [246]. The departure from the Golgi occurs by bulk flow, with no additional delay on the way to the cell periphery. Secretory vesicles of different sizes, which can carry mixed cargos of polysaccharides or polysaccharides/glycoproteins, mediate the transport of polysaccharide precursors to the cell surface [246]. Once released from the Golgi the secretory vesicles may undergo maturation, during which final cargo modifications, such as esterification of pectins or further polymerization of polysaccharides may occur [52]. However, recent experiments performed on mammalian and yeast cells [247,248] suggest that this picture of the pathway is an oversimplification, and secretion is an active process regulated by Ca^2+^-binding proteins, secretory cargo-sequestering proteins, and a TGN localized Ca^2+^ pump. Similar mechanisms could also exist in plants [62], which for a long time were thought to lack Ca^2+^-regulated exocytosis, similar to yeast. Exocytosis is a general term for denoting of the final step in the secretory pathway in which secretory vesicles fuse with the PM. The donor compartments for exocytosis are mostly (but not exclusively) the TNG, but secretory vesicles can also originate from the ER, *cis*-Golgi and MVB/PVC. The actin cytoskeleton provides a mechanism for vesicle delivery to the PM [249]. After arrival to the membrane, vesicles tether to the membrane, and after the fusion of these two membranes mediated by a SNARE complex, the content of the vesicle lumen is released to the apoplast. Tethering locates exocytotic vesicles to specific PM domains enriched in PtdIns(4,5)P_2_, and with a specific lipid content which favors membrane fusion, [250] and this process is mediated by a multisubunit complex called the exocyst. In plants homologues of all eight exocyst subunits identified in animals and yeasts (SEC3, SEC5, SEC6, SEC8, SEC10, SEC15, EXO70, EXO84) have been identified [250].

Because coalescence of the vesicles and PMs results in a net increase of membrane surface, exocytosis has to be counterbalanced by endocytosis to maintain a stable cell volume. For example, during guard cells opening their volume and the PM area increase up to about 50% due to coordinated vesicle fusion and fission [251,252,253,254]. Knowledge on the exocytosis mechanisms in plant cells is substantially less advanced compared to knowledge about endocytosis. Exocytosis in plants seems to be regulated, inducible process. The previously proposed distinction between “constitutive” and “regulated” exocytosis is misleading, at least in terms of the molecular mechanism, which appears to be basically the same in different secretion events. It is now thought that plant secretion is *always* regulated, but that fusion with the PM is not the rate-limiting step. Plant secretion is thus regulated in different time scales and is co-regulated by Ca^2+^, which is the major trigger for regulated exocytosis in animals [249]. Thus, Ca^2+^-binding proteins such as annexins can have an impact on secretion and regulate this event.

Evidence testifying that some annexin are involved in exocytosis are compelling. For example, ANXA2 is able to promote the formation of GM1/cholesterol-containing lipid microdomains corresponding to active sites of exocytosis [134]. In transmission electron microscopy (TEM), it was shown that in stimulated neuroendocrine cells ANXA2 cross-links secretory granules to the PM [255,256]. In permeabilized chromaffin cells exogenous ANXA2 can restore activity in response to Ca^2+^ [257,258].

Exocytosis appears to be driven by different secretory pathways. One of the best recognized is SNARE-mediated exocytosis. The *Arabidopsis* PM contains a define set of 23 SNAREs [259]. Among them there is nine Qa syntaxins (AtSYP111/AtKNOLLE-SYP112, AtSYP121–AtSYP125, AtSYP131–AtSYP132). Possibly they form combinatorial complexes with the other Qb SNAREs located also to the PM (AtVTI12, AtNPSN11–AtNPSN13). Besides, there are also PM-specific R SNAREs (AtVAMP721–AtVAMP722, AtVAMP724–AtVAMP726) [259].

Certain animal annexins have also been shown to interact with SNARE either indirectly, by influencing organization of sterol-enriched membrane subdomains or directly, by interacting with SNARE members of complexes. In neuroendocrine adrenergic chromaffin cells stimulation induces the membrane translocation of cytosolic ANXA2 to the PM, where it forms a heterotetramer with S100A10. In turn, S100A10 can interact with VAMP2. Enzymatic cleaves of VAMP2 solubilizes S100A10 from the plasma membrane and inhibits the translocation of ANXA2 to the plasma membrane [260]. ANXA7 associates with SNAP23 both in vitro and in vivo during surfactant secretion in alveolar cells and this process is calcium-dependent [261,262]. In addition, ANXA2 was shown to regulate secretion of lung surfactant in alveolar epithelial type II cells, which occurs by physical interaction with SNAP23 [263].

It is thought that within cells, cholesterol and sphingolipids are concentrated in the PM. Fluorescence polarization studies showed that nearly half of the plasma membrane is in ordered domains at 37 °C, and about 70–80% of the surface area of several cell types is resistant to solubilization by cold Triton X-100 [264]. In polarized epithelial cells the apical membranes specialized for secretion are almost entirely in the liquid ordered state [265]. It was shown that ANXA2 can induce formation of PI(4,5)P2-enriched domains in the plasma membrane, which possibly influence the local membrane curvature of the lipid bilayer [128].

As indicated earlier Arabidopsis annexin ANNAT4 was recently shown to interact with AtSYP121, AtSYP122, AtSYP123, AtSYP21 and AtSYP22 [190]. The functional relevance of these interactions has yet to be determined but this annexin may regulate vesicle fusion via its ability to interact with certain SNARE complexes. It is tempting to speculate that other plant annexins may interact with specific SNARE proteins and this is an important hypothesis that needs to be tested.

### 5.2. Between Golgi and Plasma Membrane: Reverse Route and Endocytosis

Just as in animal and yeast cells, endosomes in plants shuffle both biosynthetic and endocytic cargo. During l fundamental role for clathrin function in cell polarity, growth, patterning, and organogenesis in plants. Over the past decades it was shown that in plant cells endocytosis plays fundamental role in establishing cell polarity, growth, patterning, and organogenesis [266,267]. Endocytosis enables cells to dynamically control the composition and functional properties of PM, for example by internalization of receptors, which would control signaling at the PM [268]. The localization of auxin efflux transporters (PINs) is also partially regulated by endocytic recycling [269] and asymmetric localization of PINs determines cell polarity and promotes the directionality of intercellular auxin flow [23]. Interestingly, expression of ANNAT2 in Arabidopsis root cap columella cells is altered in *pin2* mutants in response to hypergravity [270]. The authors of this study suggest that PIN2 may be responsible for normal expression and localization of ANNAT2 during gravity responses. In plant cells endocytosis also participates in the formation of the cell plate [271]. Membrane proteins and apoplastic fluids are constantly retrieved by budding, scission and formation of endocytic vesicles, which fuse with endosomes giving early endosomes (EE). In plants the TGN functions as early endosomes receiving the exocytosed cargo [46]. The TGN functions as an early endosome and receives internalized endocytotic vesicle [48,272,273].

Endocytosis is a precisely regulated process and its molecular mechanisms are tightly controlled as well as separated from another transport routes. An Arabidopsis mutant that is lacking the TGN-located component and defective in the secretory pathway displays no obvious impairment in endocytotic trafficking [274]. The elements of endocytotic machinery, i.e., lipids and appropriate receptors, are recycled back to the PM with recycling endosomes, whereas other cargo proteins undergo sorting and are delivered by late endosomes to the lytic vacuole for degradation [275,276].

Endosomes can be classified according to structural features into tubular or multivesicular, or by functional criteria into sorting (SE) or recycling (RE) [277,278]. In general, transport through the endosomal system relies on maturation accomplished by component replacement. This process involves removal of the remnants of the previous traffic phase and the parallel introduction of new ones, thus gaining the competence to execute the next step in trafficking. Additionally, maturation of endosomes is accompanied by acidification of the internal lumen due to the increasing amounts and activity of the membrane V-ATPase (vesicular H^+^-ATPase subunit a1) [279,280,281]. In humans, disturbances in this process appear as a major cause of numerous neurodegenerative diseases [180].

Just as in animal and yeast (*Saccharomyces cerevisiae*) cells, several types of endocytosis have also been described also in plants. The primary difference in endocytosis between plant and animal cells is the mechanism of vesicle formation, which impacts both vesicle size and the material that is incorporated into the vesicles. These differences include fluid phase uptake, phagocytosis of bacteria, and lipid raft-mediated endocytosis [182]. Two types of endocytosis have been documented: clathrin-mediated and membrane microdomain-associated [44,282,283]. Regardless of the type of endocytosis, vesicles are fused with an internal membranous compartment.

### 5.3. Multivesicular Body/Prevacuolar Compartment

MVBs are formed by the fusion of Golgi-derived vesicles carrying newly synthesized vacuolar proteins as well as from early/recycling endosomal compartments. Within MVB/PVC two kinds of sorting processes occur—the recycling of vacuolar cargo receptors mediated by the retromer complex and the sorting of PM protein into internal vesicles by the ESCRT machinery [284]. Fusion of MVBs/PVCs with the vacuole leads to the release of soluble vacuolar proteins and MVB vesicles into the lumen of the vacuole [191,274]. Thus, MVBs/PVCs serve as intermediate compartments that enable proteins to recycle before their fusion with the vacuole.

The characteristic feature of MVB/PVC is the presence of intraluminal vesicles (ILV). In yeast and mammals, these vesicles are thought to function as late endosomes. Generation of ILVs is coordinated by the conserved ESCRT machinery (ESCRT-0, -I, -II, -III and the VPS4 complexes) [285]. Plant genomes encode orthologs of three ESCRT-I, II and III complexes but lack ESCRT-0 [286,287,288,289]. So the other proteins, e.g., FREE1 (plant-specific and PVC-localized FYVE domain protein required for endosomal sorting) [290] and TOM1 (target of MYB) contribute to the cargo recognition [41]. RAB GTPase-mediated MVB/PVC maturation occurs by component replacement. In Arabidopsis maturation of MVB/PVC is connected with gradual replacement of Rab5 by Rab7 to form a Rab7-positive MVB/PVC. As well, fusion between MVB/PVC and autophagosomes has been observed [291].

It was shown that in Arabidopsis ANNAT3 is required for the TGN-to-MVB transport. In protoplasts from plants with suppressed expression of ANNAT3 by RNAi, maturation of MVB was disturbed, which was manifested by increased co-localization of TGN and MVB markers (YFP-AtSYP61 and mRFP-AtVSR2). Moreover, RNA interference-mediated knockdown plants (Δ*annat3*) had the same phenotype as the dominant-negative mutant of VPS2, which is a member of ESCRT-III complex required for the fission of the internal vesicles in MVBs. Taken together, these results suggest that ANNAT3 is necessary for the final step of releasing MVBs as a transport carrier to the vacuole [191].

### 5.4. Between Plasma Membrane and Vacuoles

Another specificity of the plant endomembrane system, contributing an additional level to the trafficking system complexity, is the presence of different types of vacuoles in the same cells. In most mature plant cells the vast majority of the cellular volume is occupied by a large central vacuole. It provides structural support for plant cells by exerting turgor pressure against cell wall, and it drives cell expansion without the necessity to produce more cytosol. Several early studies characterized a vacuolar-specific annexin, VCaB42. This annexin binds calcium in the low nanomolar range [162], co-localizes with a Rop GTPase and is suggested to function in vacuolar biogenesis during cell expansion [292]. Central vacuoles also serve as the site for an intracellular storage of water and nutrients, waste and toxins, and as sequesters of non-active precursor forms of proteins/secondary compounds essential for interactions with the environment or other organisms [42]. Vacuoles may also support other very specialized functions, for example in guard cells, where the rapid splitting and fusion of vacuoles enable fast changes in cell volume and stomatal movement [293].

In young cells, especially during seed development, there are at least two functionally distinct types of vacuoles [294,295] -PSVs and -LVs. PSVs have a neutral pH and are responsible for storage of nutrients and proteins indispensable for germination and early seedlings growth, whereas the LVs are acidic and dedicated to protein degradation [49,296,297]. PSV and LV are believed to be separate organelles [294,298,299,300]. During seed germination, cell type-specific transformation of PSV into the LV can occur and PSVs are rapidly replaced by a central LV to enable rapid elongation of embryo cells [301]. In vegetative cells, e.g., in a growing root tip, the fusion of the PSV and the LV appear to occur [294]. Transport of proteins to vacuoles originates predominantly from the TGN but can also begin directly from the ER. Such a shortcut possibly represents a rapid mechanism necessary for continuous adaptation to a changing environment and adaptation to stress [302]. The LV and PSV are the final stations of the vesicular traffic. For their proper shaping an undisturbed post-Golgi trafficking is required.

Overall, scientists are only beginning to understand the details of how the plant TGN recognizes and segregates the proteins to different vacuoles [62,303]. During cellular trafficking, proteins are targeted to defined compartments based on the presence of special integral motifs recognized by compatible receptors. Vacuolar sorting signals (VSSs) that operate in plants can be divided into three main groups. First is sequence-specific vacuolar sorting signal (ssVSR) found frequently in the LV directed protein; i.e., the N-terminal NPIR (Asn-Pro-Ile-Arg) consensus sequence that is very strict and does not tolerate variations. This sequence is usually a part of longer propeptides cleaved off during protein maturation in the PVC or in the vacuoles.

For a PSV targeted protein a non-sequence-specific cleavable C-terminal propeptide (CTPP) has been described. It is a C-terminal sequence with no clear consensus of sequence or length, but usually enriched in hydrophobic amino acids. Finally, there are signals dependent on the tertiary structure of the molecule, mostly common in storage proteins. These tertiary structures may be distributed throughout the molecule and achieve functionality only when the protein acquires its native conformation [297,304]. The VSSs are recognized by sorting receptors responsible for directing proteins to the vacuoles. So far, two types of such receptors have been described, vacuolar sorting receptors (VSRs) [305,306] and receptor homology-transmembrane-RING H2 domain proteins (RMRs) [307,308].

The main route for trafficking of soluble cargo from the Golgi to the LV requires recognition of ssVSS by VSRs at the TGN, and transport to the PVC/MVB [306] by clathrin-coated vesicles before reaching the vacuoles [302]. When vesicles fuse with PVC/MVB the VSRs are recycled back to the TGN for the next round of delivery. When PVC/MVB fuse with the tonoplast, cargo molecules are released to the vacuole [65]. Such step-by-step traffic supported by the sequential action of Rab GTPases, with Rab11 mediating early transport events and the arrival of cargo at the PVC, while Rab7 mediates the final delivery to the vacuole and increases cargo levels in PVCs [207].

In contrast to soluble proteins, the sorting signals for tonoplast spanning proteins are largely unknown. At least three different pathways exist, and membrane proteins can reach the vacuoles even when Golgi and post-Golgi trafficking is blocked [63,66,309,310], possibly with the aid of autophagy machinery [311,312]. In plants, proteins with the single TMD located in the PVC contain the Yxxφ motif at their C-terminal cytosolic domain [313]. However, the sorting motifs for multi-TMD proteins remain entirely unknown.

The transport of VSS-VSR complexes from the TGN is presently thought to be a passive process that relies on release of TGN from the Golgi stack and its subsequent maturation into a MVB/PVC, in a way similar to that of endosomes. Hence, only one type of CCV is produced that recycles membrane proteins back to the PM [60,61,62,314].

In Arabidopsis 9 SNAREs locate to the tonoplast, namely AtSYP21 and AtSYP22 (Qa), AtVTI11 and AtVTI13 (Qb), AtSYP51 and AtSYP52 (Qc), and AtVAMP711, AtVAMP712 and AtVAMP713(R). ANNAT4 interacts with two of these SNARES, AtSYP21 and AtSYP22, and thus is a candidate for regulating vesicle fusion with vacuolar membranes. Only one complete SNARE complex has been identified and confirmed to be involved in vacuolar trafficking, and it is an endosomal type, namely (Qa/Qb/Qc/R) AtSYP22/AtVTI11/AtSYP51/AtVAMP727 [315]. Members of SYP5-subgroup (AtSYP51 and AtSYP52) interact specifically with a syntaxins of SYP2-subgroups, as well as with the AtVTI11 to form a SNARE complex involved in the TGN-to-PVC trafficking [316].

### 5.5. Leaderless Secretion

Secretion of proteins lacking the N-terminal signaling sequence that do not enter the classical ER-mediated secretion pathway in animals is well accepted. Four general mechanisms have been proposed: (i) direct translocation across the PM (e.g., fibroblast growth factor 1, interleukin 1α); (ii) endolysosomal pathways in which cytosol proteins are transported into intracellular vesicles called endolysosomes via protein-conducting channels (IL-1β and HMBG1); the fusion of endolysosomes with the cell membrane and release of the proteins into the apoplast; (iii) exosome-mediated secretion; and (iv) membrane blebbing or microvesicle shedding [317].

Annexins do not contain N-terminal signaling sequences in their primary amino acid sequence directing them to be secreted, however they have been detected in phloem sap and in the extracellular matrix in plants [318,319,320,321]. Extracellular vesicles/exosomes have been identified in plants and like animal exosomes have been suggested to function in part as a novel way for proteins without signal peptides to be secreted [322]. Interestingly, the presence of ANNAT1 and other membrane trafficking proteins including AtSYP121 and AtSYP122 was recently documented in Arabidopsis exosomes [323].

Recently, non-classical secretion was shown to be rapidly modified in response to biotic stress. Upon induction with salicylic acid, the secretion of a large number of usually cytosolic enzymes that lack a classical signal peptide, such as superoxide dismutases, occurs soon after the salicylic treatment occurs [321,324,325].

## 6. Future Perspectives-Potential Mechanisms of Annexins’ Effect on Cellular Trafficking

**Figure 3:**
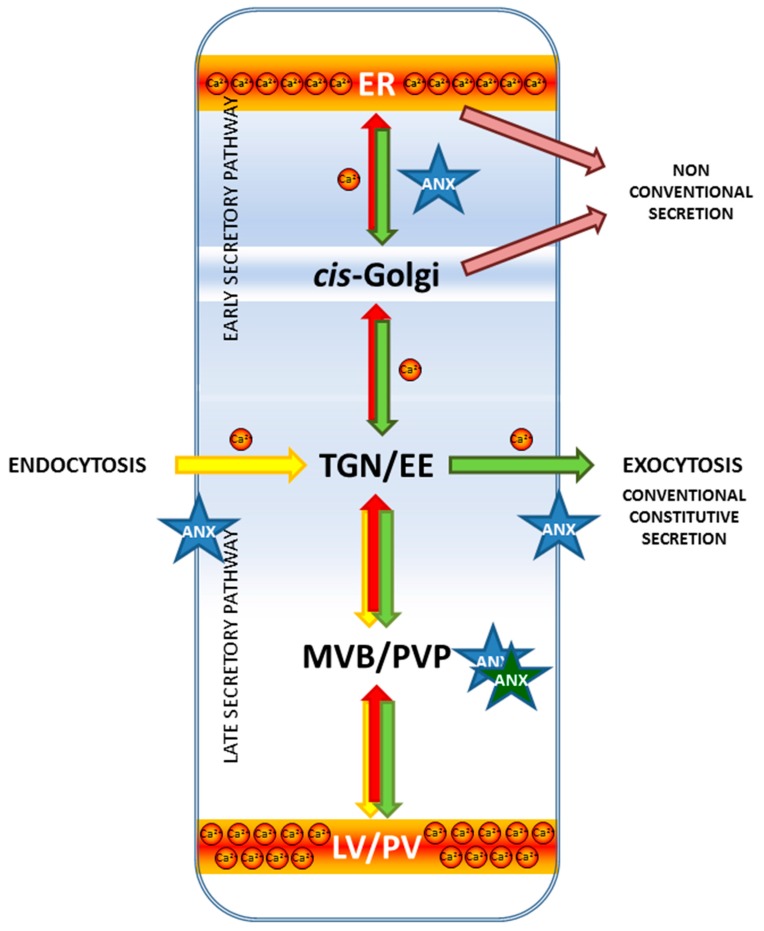
Potential targets for annexin participation in intracellular transport in plant cells. There is evidence that annexins participate in both the early and late secretory pathways including endo- and exocytosis. Annexins are also suggested to function in conventional constitutive secretion as well as non-conventional secretion; green arrow—forward route, red arrow—reverse route; yellow arrow—endocytic route.

Although there is strong evidence for the involvement of annexins in plant membrane trafficking, thus far there is not much known about the details of annexin function in specific membrane trafficking events, with the notable exception of evidence that ANNAT3 functions in post-TGN vesicular transport of soluble vacuolar proteins [191]. In general, it appears that plant annexins play important roles in the ESP as well as in the LSP, including post-Golgi trafficking (Figure 3). Because annexins bind membranes in response to an increase in cytosolic Ca^2+^ levels, they may act similarly to the Ca^2+^ sensor, calmodulin, with the main difference that they transduce the Ca^2+^ signal into membrane structures and in this way may act as a Ca^2+^-dependent activators for secretion.

It is also possible that certain plant annexins could modulate specific endomembrane vesicle fusion events by modifying the pH and membrane potential of endomembrane vesicles. Future research should test the specific membrane and protein interactions of individual annexins in order to gain a better understanding of the contribution of annexins to plant membrane trafficking.
